# Reply; Testotoxicosis: Report of Two Cases, One with a Novel Mutation in LHCGR Gene

**DOI:** 10.4274/jcrpe.2765

**Published:** 2016-03-01

**Authors:** Olcay Evliyaoğlu

**Affiliations:** 1 İstanbul University Cerrahpaşa Faculty of Medicine, Department of Pediatric Endocrinology, İstanbul, Turkey

**Keywords:** testosterone, LHCGR gene, Novel mutation

## DEAR EDITOR

I have reviewed the letter to the editor regarding “Testotoxicosis: Report of Two Cases, One with a Novel Mutation in LHCGR Gene”.

I appreciate authors’ interest and contributions to our report about 2 cases with LHCGR gene mutations. We have reported two cases with similar clinical features suggesting peripheral precocious puberty, one with a novel and one with a known mutation in LHCGR gene leading to activation of the receptor. It is well known that the management of these patients is quite difficult because of uncontrolled testosterone secretion from the testes which is the main reason for precocious pubertal signs. We initially started bicalutamide and anastrozole treatments in both of our patients, as bicalutamide treatment had been tried in similar cases as a potent antiandrogen (1). However, in the follow-up, bone age advancement continued rapidly with the pubertal progression in both patients. Additionally, serum testosterone levels were still in high range (~400-800 ng/dL) without significant decline. We changed our treatment regimen to ketoconazole and anastrozole because we could not cease the pubertal and bone age advancement that were also associated with high testosterone levels. Unequivocally, we treat the patient not the laboratory results, but we cannot deny the testosterone effect on the clinic. Testosterone is responsible for the appearance of secondary sexual characteristics, whereas estrogen is the hormone responsible for the epiphyseal maturation which is converted from testosterone by aromatization. High testosterone levels are associated with bone age advancement. Evaluation of pubertal progression and bone age advancement associated with serum testosterone levels are important indicators for treatment monitoring (2,3).

In conclusion, we could not get any benefit from bicalutamide treatment in our patients, whereas ketoconazole treatment is promising in short term. Overall, ‘successful treatment’ can only be evaluated in long-term follow-up of these patients.

Financial Disclosure: The authors declared that this study has received no financial support.
